# Proliferation in cardiac fibroblasts induced by β_1_-adrenoceptor autoantibody and the underlying mechanisms

**DOI:** 10.1038/srep32430

**Published:** 2016-08-31

**Authors:** Tingting Lv, Yunhui Du, Ning Cao, Suli Zhang, Yulin Gong, Yan Bai, Wen Wang, Huirong Liu

**Affiliations:** 1 Department of Physiology and Pathophysiology, School of Basic Medical Sciences, Capital Medical University, Beijing 100069, PR China; 2 Beijing Key Laboratory of Metabolic Disorders Related Cardiovascular Disease, Capital Medical University, Beijing 100069, PR China

## Abstract

Chronic sustained stimulation of β-adrenoceptor is closely related to cardiac fibrosis which is bad for cardiac function. Growing evidence showed that the high prevalence of β_1_-adrenoceptor autoantibody (β_1_-AA) in the sera of patients with various types of cardiovascular diseases decreased cardiac function. In the current study, we demonstrated that β_1_-AA impaired the cardiac function evaluated by echocardiography and that β_1_-AA triggered cardiac fibrosis in terms of increased expression of α-smooth muscle actin as the marker of myofibroblast and collagen deposition in a passive β_1_-AA immunized mice model during 16 weeks. Further, we showed that β_1_-AA activated β_1_-AR/cAMP/PKA pathway and promoted proliferation in primary cardiac fibroblasts through specific binding to β_1_-AR but not to β_2_-AR. Moreover, β_1_-AA was also likely to promote proliferation in cardiac fibroblasts through activating p38MAPK and ERK1/2 as p38MAPK inhibitor SB203580 and ERK1/2 inhibitor PD98059 partially reversed the proliferative effect. The persistent activating signalling of PKA and P38MAPK in 1 h induced by β_1_-AA was associated with lacking agonist-induced desensitization phenomena. The conditioned medium from β_1_-AA-stimulated cardiac fibroblasts induced cardiomyocyte apoptosis, which indicated that β_1_-AA changed the secretion of cardiac fibroblasts contributing to cardiac injury. These findings will contribute to our understanding of the pathological mechanisms of β_1_-AA.

Despite advances in medical treatment level, cardiovascular disease remains a major public health issue with high morbidity and mortality worldwide. Cardiac fibrosis characterized by excessive collagen deposition^1^ is associated with various types of cardiovascular diseases such as heart failure, arrhythmia, and cardiac sudden death. It has been reported that the presence and amount of myocardial fibrosis impacted on arrhythmic outcome and sudden cardiac death in patients with nonischemic dilated cardiomyopathy^2^. However, the pathogenesis involved in cardiac fibrosis is not yet well understood. Accumulated evidence indicated that the overstimulation of β-adrenergic receptor (β-AR) induced adverse myocardial remodeling^3^ and treatment with β_1_-selelctive antagonist was beneficial to cardiac function and structure^4^. For instance, previous studies reported that cardiac fibrosis occurred by chronic stimulation with a β_1_/β_2_-adrenoceptor agonist isoprenaline (ISO) or in β_1_-adrenoceptor transgenic mice^5^. However, the etiology of cardiac fibrosis induced by β_1_-adrenoceptor has not been revealed completely.

Recently, a large number of clinical investigations have proved that β_1_-adrenoceptor autoantibody (β_1_-AA) was found for high frequency and titers in the sera from patients with dilated cardiomyopathy^6^, chagas disease^7^. heart failure^8^,^9^ and other diseases^10^. Accumulated evidence indicated that β_1_-AA recognized β_1_-adrenoceptor and subsequently activated cyclic adenosine 3′,5′-monophosphate (cAMP)/protein kinase A (PKA), exerting agonist-like effects such as positive inotropic and chronotropic effects^11^,^12^,^13^. More interestingly, β_1_-AA-induced increase in beating frequency of neonatal cardiomyocytes remained unchanged for more than 6 h lack of desensitization while ISO underwent desensitization within 60 minutes^13^. Badly, long term stimulation with β_1_-AA led to cardiac dysfunction^14^,^15^, and even increased the susceptibility to arrhythmia^16^,^17^ as well as sudden cardiac death^18^,^19^. In particular, our previous data showed that the positive rate and titer of β_1_-AA were significantly higher in models of heart failure established by abdominal aortic coarctation or doxorubicin injection when myocardial fibrosis simultaneously occurred^20^. However, the influence of β_1_-AA on myocardial fibrosis remains little.

Cardiac fibroblast, as the most abundant cell type among the non-cardiomyocytes in the heart, plays numerous roles in cardiac development and remodeling^21^. The proliferation and secreting excessive extracellular matrix protein of cardiac fibroblast is concentrated on contributing to cardiac fibrosis^22^. Previous evidence showed that p38MAPK^23^ and ERK1/2^24^ were involved in proliferation of cardiac fibroblasts. Our previous work suggested that β_1_-AA enhanced proliferation and secretion of lymphocytes through activating β_1_-AR/cAMP/PKA and p38MAPK^25^. Others’ research reported that β_1_-AA activated ERK1/2 in cardiomyocytes^26^. These experimental results, taken together with the expression of β_1_-AR on the surface of cardiac fibroblasts^27^, suggested that β_1_-AA may be responsible for cardiac fibroblasts proliferation.

This study was designed to establish a passive β_1_-AA immunized mice model to investigate the effect of β_1_-AA on myocardial fibrosis *in vivo* and to determine the impact of β_1_-AA on the proliferation and the underlying mechanisms in cultured cardiac fibroblasts *in vitro*. Our data will provide new experimental evidence for pathological mechanisms of β_1_-AA.

## Results

### Cardiac fibrosis occurred in a passive immunization mouse model with monoclonal antibodies of β_1_-AR EC_II_

In order to obtain sufficient β_1_-AA, a monoclonal antibody against the second extracellular loop of β_1_-AR (β_1_-AR mAb) was synthesized by hybridoma technique and purified from the ascites of BALB/c mice using a MabTrap kit. It was clearly displayed that synthesized β_1_-AR mAb had the IgG heavy chains (the relative molecular mass of about 55 KD) and IgG light chains (the relative molecular mass of about 25KD) (Fig. S1A). Moreover, concentration of β_1_-AR mAb detected by SA-ELISA was much higher compared to β_1_-AA-negative IgG (Fig. S1B). As shown in Fig. S1C, primary cultured cardiomyocytes from neonatal rat hearts were isolated successfully and identified by positive α-actinin staining. The spontaneous beating frequency of primary cultured neonatal rat cardiomyocytes was remarkly increased by stimulation of β_1_-AR mAb, which was blocked by β_1_-AR blocker metoprolol and β_1_-AR EC_II_ peptide (Fig. S1D). Our experimental results indicated that activated β_1_-AR mAb was successfully synthesized, which could be used to the following study.

**Figure 1:**
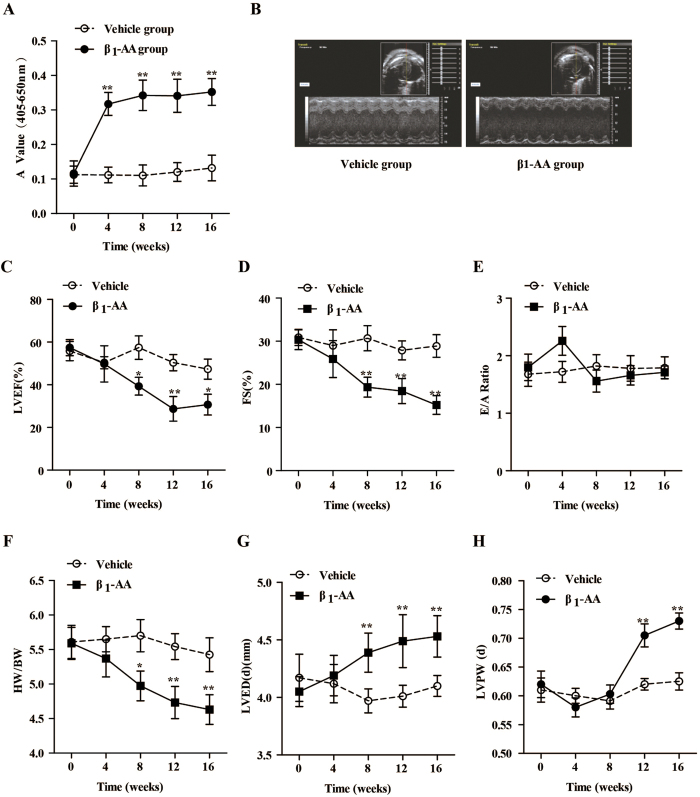
Cardiac function in vehicle and β_1_-AA positive mice model during treated 16 weeks. (**A**) The level of β_1_-AA at different time points during the treated 16 weeks. (**B**) Images are representative of echocardiogram at the 16^th^ week. (**C–H**) The LVEF % (left ventricular ejection fraction), FS% (fraction shortening), E/A ratio, heart weight/Body weight (HW/BW), left ventricular end diastolic dimension (LVEDd, mm) and left ventricular posterior wall end-diastolic thickness (LVPWT, mm) of β_1_-AA positive group and vehicle group mice during the 16 weeks. Data are presented as mean ± SEM. ^*^P < 0.05 *vs.* vehicle group, ^**^P < 0.01 *vs.* vehicle group, n = 8/group at different time points.

To create a passive immunization mouse model, synthesized β_1_-AR mAb (hereinafter referred to β_1_-AA) was intraperitoneally injected into the BALB/c mouse at a dose of 5 μg/g body weight once every two weeks during 16 weeks. Our results showed that the level of β_1_-AA remained in the BALB/c mice over time, as indicated by ELISA, suggesting that a passive immunization mouse model with β_1_-AA was established successfully (Fig. 1A). Cardiac function in β_1_-AA and vehicle mice was assessed using M-mode echocardiography. At the 16^th^ week of passive immunization, left ventricular chamber of the mice enlarged in β_1_-AA group (Fig. 1B). The left ventricular ejection fraction (EF) and fractional shortening (FS) began to decrease at the 8^th^ week, and then continued to decrease at the 12^th^ and 16^th^ week (Fig. 1C,D). The results indicated that the systolic cardiac function of β_1_-AA-treated mice was remarkly decreased. Early diastolic and late diastolic mitral flow velocity ratio (E/A Ratio) in β_1_-AA group unchanged during the 16 weeks (Fig. 1E), which suggested that the diastolic cardiac function did not change obviously. All the data suggested that β_1_-AA alone reduced the cardiac function in the mouse model. We also assessed heart structure of the β_1_-AA positive model. As shown in Fig. 1F–H, the ratio of heart weight and body weight began to decrease at the 8^th^ week and decreased more significantly at the 12^th^ and 16^th^ week (Fig. 1F). Left ventricular end-diastolic diameter (LVEDd for short) and left ventricular posterior wall end-diastolic thickness (LVPWT for short) assessed by echocardiography were both enlarged in β_1_-AA group than that in vehicle group at week 12^th^ and 16^th^ (Fig. 1G,H).

**Figure 2:**
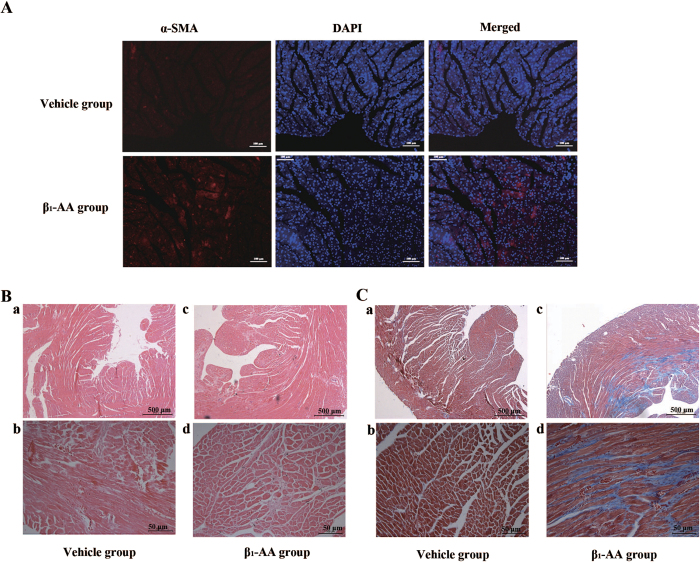
Cardiac fibrosis occurred in β_1_-AA positive mice. (**A**) The expression of α-smooth muscle actin (α-SMA) in the heart of vehicle and β_1_-AA group at the 8^th^ week of model. (**B**) The morphology of heart in vehicle and β_1_-AA group determined by HE staining at the 16^th^ week of model. (**C**) Collagen deposition of heart in vehicle and β_1_-AA positive group detected by Masson Trichrome staining at the 16^th^ week of model. Scale bar = 500 or 50 μm, respectively.

Moreover, as early as 8 weeks, the expression of α-SMA in mice heart of β_1_-AA group was much higher than that in vehicle group (Fig. 2A), which highly predicted the phenotypic change of fibroblasts to myofibroblasts contributing to cardiac fibrosis. At the same time, the collagen deposition appeared in the heart of β_1_-AA group (Supplementary Information Fig. S2). At the 16^th^ week of passive immunization, HE staining showed increased cardiac interstitial and Masson trichrome staining showed greater fibrosic areas with increased collagen deposition in β_1_-AA group than that in vehicle group (Fig. 2B,C), which indicated that β_1_-AA induced cardiac fibrosis. The above-mentioned results indicated that the long term presence of β_1_-AA triggered the cardiac fibrosis.

### β_1_-AA promoted proliferation in primary cardiac fibroblasts

**Figure 3:**
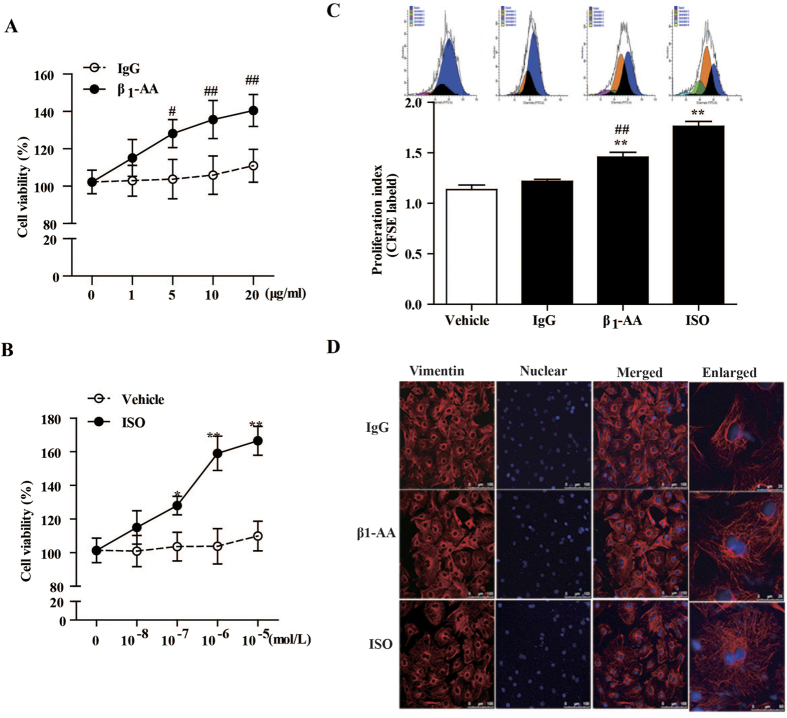
β_1_-AA increased cell viability of cardiac fibroblasts (CFs). (**A,B**) Different concentrations of β_1_-AA and β_1_/β_2_-AR agonist isoprenaline (ISO) were added to cultured cardiac fibroblasts respectively for 24 h, then the cell viability was detected by Cell Counting Kit-8. n = 6 per group. (**C**) CFs before different treatments were labeled with 5 μmol/ml CFSE and cell proliferation was measured by flow cytometry. Data shown here are representative of one of three individual experiments with similar results. The bar graph showed the statistical results. (**D**) The morphology of cardiac fibroblasts treated with β_1_-AA and ISO. Vimentin, which is a marker of cardiac fibroblasts, was detected using immunofluorescence and observed in confocal microscopy. After β_1_-AA and ISO were added to cardiac fibroblasts and allowed to incubate for 24 h, the morphology of cardiac fibroblasts changed. The cells had more cytoplasm and there were more two-nucleus cells. All data are presented as the mean ± SEM of three independent experiments. ^**^P < 0.01 *vs.* vehicle group, ^##^*P* < 0.01 *vs.* IgG group. (β_1_-AA negative IgG). IgG: β_1_-AA-negative mouse IgG.

To determine the influence of β_1_-AA on proliferation in primary cardiac fibroblasts, we isolated cardiac fibroblasts from neonatal rat heart and detected the effect of β_1_-AA on proliferation of primary cardiac fibroblasts by Cell Counting Kit-8 (CCK-8) and carboxyfluorescein diacetate succinimidyl ester (CFSE) staining. The positivity of cardiac fibroblasts identified by immunofluorescence cellular staining of vimentin was more than 95% (Fig. S2). The results detected by CCK-8 were as follows: compared to a non-specific IgG group (β_1_-AA negative), 5, 10, and 20 μg/ml of β_1_-AA all markedly increased cell viability of fibroblasts at 24 h while lower concentration 1 μg/ml of β_1_-AA had no proliferation effect (Fig. 3A); positive control β_1_-AR/β_2_-AR isoprenaline (ISO for short, 10^−7^, 10^−6^, and 10^−5 ^mol/L) increased cell viability of cardiac fibroblasts in a concentration-dependent manner at 24 h (Fig. 3B). The results showed that 10 μg/ml β_1_-AA or 10^−6 ^mol/L ISO obviously promoted proliferation of cardiac fibroblasts. Thus, 10 μg/ml β_1_-AA or 10^−6 ^mol/L ISO were used to explore the specific proliferation mechanisms in the following study. Indeed, analysis of CFSE staining by flow cytometry clearly showed that the proliferation index of cardiac fibroblasts treated with either 10 μg/ml β_1_-AA or 10^−6 ^mol/L ISO for 24 h was elevated in cardiac fibroblasts than that of cardiac fibroblasts treated with the non-specific IgG or vehicle respectively (Fig. 3C). Additionally, cell morphology was observed by immunofluorescence cellular staining of vimentin using a laser scanning confocal microscope. As shown in Fig. 3D, cardiac fibroblast which had two or three nucleus increased after stimulation with either β_1_-AA or ISO for 24 h, suggesting that β_1_-AA enhanced proliferation of cardiac fibroblasts.

### β_1_-AA bound to β_1_-AR in cardiac fibroblast directly

**Figure 4:**
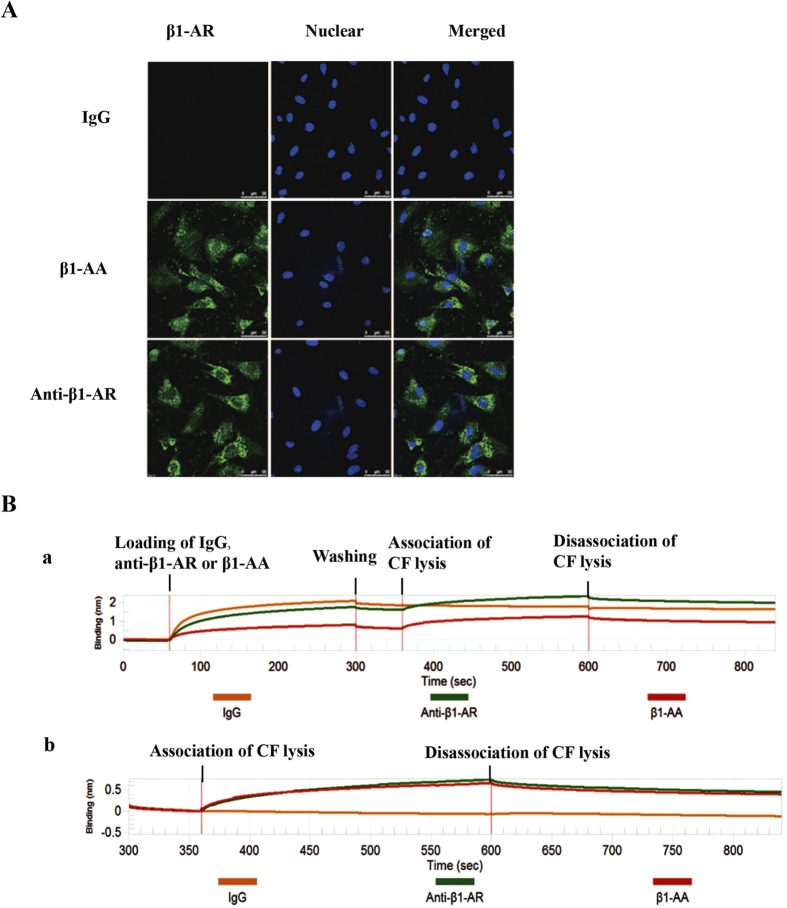
β_1_-AA bound to β_1_-AR in cardiac fibroblasts. (**A**) The binding of β_1_-AA/purchased anti-β_1_-AR primary antibody to β_1_-AR by immunfluorescence cellular staining. (**B**) The BLItz system results for the interaction of β_1_-AA-negative mouse IgG (yellow line), β_1_-AA (red line) and purchased anti-β_1_-AR primary antibody (green line) with β_1_-AR in CFs are shown. The vertical and horizontal axes represent the light shift distance (nm) and association/dissociation time (sec), respectively.

To explore the underlying mechanisms of β_1_-AA on proliferation of cardiac fibroblasts, we analyzed the direct binding of β_1_-AA and β_1_-AR in cardiac fibroblasts by immunofluorescence cellular staining, immunoprecipitation and BLItz system. Results showed that β_1_-AA used as the primary anti-β_1_-AR in the present study bound to β_1_-AR on the surface of cardiac fibroblasts directly, while non-specific IgG did not (Fig. 4A). Immunoprecipitation results displayed that β_1_-AA as well as a purchased β_1_-AR primary antibody interacted with β_1_-AR in cardiac fibroblast, while non-specific IgG did not bind to β_1_-AR (Fig. 4B). In addition, BLItz system further confirmed the direct binding of β_1_-AA and β_1_-AR. The kd value of purchased anti-β_1_-AR and β_1_-AA binding to cardiac fibroblasts were 3.411e^−3 ^mol/L and 3.455e^−3 ^mol/L respectively while the kd value of non-specific IgG binding to cardiac fibroblasts was less than 1e^−7 ^mol/L. The data showed that the affinity of β_1_-AA (or a purchased anti-β_1_-AR antibody) and cardiac fibroblast were both much higher than non-specific IgG, which significantly suggested the specific binding of β_1_-AA and β_1_-AR in cardiac fibroblasts (Fig. 4C).

### β_1_-AA promoted proliferation in cardiac fibroblast partially through β_1_-AR/cAMP/PKA

**Figure 5:**
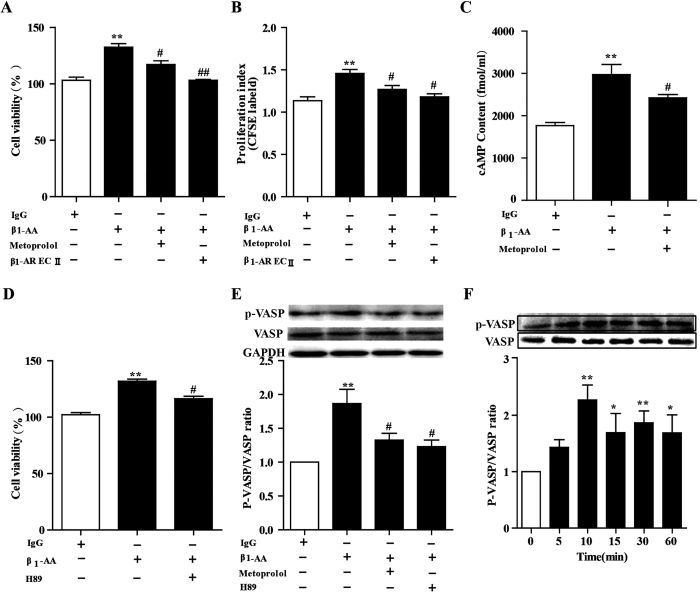
β_1_-AA induced the proliferation of cardiac fibroblasts (CF) partially via the β_1_-AR/cAMP/PKA pathway. (**A**) CF was pretreated with the selective β_1_-AR antagonist metoprolol (1 μmol/ml) for 30 min before adding β_1_-AA (10 μg/ml). Cell viability of CF was detected by CCK8 assay. (**B**) Increased proliferation index induced by β_1_-AA was partially reversed by β_1_-AR antagonist metoprolol by CFSE labeling. (**C**) The intracellular cAMP concentration of CF was determined by [^125^I] cAMP RIA KIT in different treatments. (**D**) Cell viability of CFs were pretreated with the PKA inhibitor H89 (1 μmol/ml) for 30 min before adding β_1_-AA (10 μg/ml). (**E**) Immunoblot detection of phosphorylated VASP (p-VASP) and total VASP form CFs stimulated with β_1_-AA (10 μg/ml) for 30 min. Images are representative of 3 independent repeated experiments. The bar graph shows the ratio of p-VASP to total VASP. (**F**) Immunoblot detection of phosphorylated VASP (p-VASP) and total VASP form CFs stimulated with β_1_-AA (10 μg/ml) for different time point in 60 min. n = 3 per group. ^**^P < 0.01 *vs.* vehicle (PBS) group, ^##^*P* < 0.01 *vs.* β_1_-AA group. VASP: vasodilator-stimulated phosphoprotein; P-VASP: phospho-vasodilator-stimulated phosphoprotein.

β_1_-AR/cAMP/PKA is the classical signaling of exerting physical and pathological effects in cardiac cells. PKA-substrate vasodilator-stimulated phosphoprotein (VASP) is a key effector of cAMP, whose principal function is regulated by PKA via phosphorylation at Ser^157^ 28. To determine whether the β_1_-AR/cAMP/PKA pathway was involved in proliferation of cardiac fibroblast induced by β_1_-AA, cardiac fibroblasts were pretreated with the β_1_-selective antagonist blocker metoprolol or the PKA inhibitor H89 for 30 min before treatment with β_1_-AA. Results showed that metoprolol partially blocked the proliferation effects of β_1_-AA on cardiac fibroblasts and that the supernatant from incubation of β_1_-AA and β_1_-AR EC_II_ reversed the effects completely (Fig. 5A), which suggested β_1_-AA promoted proliferation of cardiac fibroblasts partially through β_1_-AR. Similarly, CFSE staining assay showed that the cell viability of cardiac fibroblasts stimulated by β_1_-AA was partially reversed by pretreatment with metoprolol or β_1_-AR EC_II_ (Fig. 5B). In addition, β_1_-AA increased the concentration of intracellular cAMP of cardiac fibroblasts, which was blocked by metoprolol partially (Fig. 5C). Pretreatment with PKA (downstream of cAMP) inhibitor H89 for 30 min before adding β_1_-AA also partially reversed the increased cell viability induced by β_1_-AA (Fig. 5D). PKA activity monitored by ratio of p-VASP/VASP protein in cardiac fibroblasts increased by stimulation with β_1_-AA, which was reversed partially by pretreatment of metoprolol or H89 (Fig. 5E). Interestingly, β_1_-AA persistently activated PKA activity in 1 h which is different from agonist-induced desensitization (Fig. 5F). All of these results indicated that β_1_-AA stimulated cardiac fibroblasts proliferation at least partially through the β_1_-AR/cAMP/PKA pathway.

### Involvement of β_2_-AR in β_1_-AA-mediated proliferation in cardiac fibroblast

**Figure 6:**
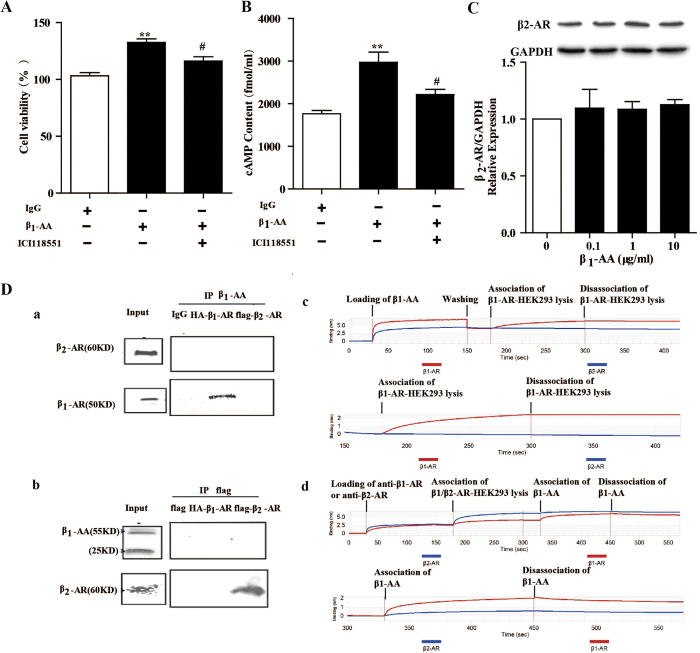
β_1_-AA might promote proliferation in cardiac fibroblast (CF) via β_2_-AR partially. (**A**) CFs were pretreated with the selective β_2_-AR antagonist ICI118551 (1 μmol/ml) for 30 min before treatment with β_1_-AA (10 μg/ml). Cell viability of CF were detected by CCK8 assay. ^**^P < 0.01 *vs.* IgG group, ^#^*P* < 0.05 *vs.* β_1_-AA group.^##^*P* < 0.01 *vs.* β_1_-AA group. (**B**) The intracellular cAMP concentration of CF was determined by [^125^I] cAMP RIA KIT in different treatments. ^**^P < 0.01 *vs.* IgG group, ^#^*P* < 0.05 *vs.* β_1_-AA group.^##^*P* < 0.01 *vs.* β_1_-AA group. (**C**) CFs were stimulated with β_1_-AA (0, 5, 10, 20 μg/ml) for 24 h, then β_2_-AR relative expression were unchaged by western blotting. ^*^P < 0.05 *vs.* vehicle group (0 μg/ml). (**D**) HA -β_1_-AR and flag-β_2_-AR were overexpressed on HEK293 cells by plasmid transfection. β_1_-AA interacted with HA-β_1_-AR but not flag-β_2_-AR by immunoprecipitation (a and b). The binding of β_1_-AA and β_1_-AR/β_2_-AR was detected by BLItz system (c and d). β_1_-AA bound to cell lysis of β_1_-AR overexpressed HEK293 cells, but not cell lysis of β_2_-AR overexpression-HEK293 cells (**C**). β_1_-AA bound to cell lysis of β_1_-AR overexpressed-HEK293 cells which were pre-bound to purchased anti-β_1_-AR, but not cell lysis of β_2_-AR overexpressed HEK293 cells which were pre-bound to purchased anti-β_2_-AR (**D**). Data shown here are representative of one of three individual experiments with similar results.

β_2_-AR plays a significant role in proliferation of cardiac fibroblasts. To examine whether β_2_-AR was involved in fibroblasts proliferation induced by β_1_-AA, specific β_2_-AR blocker ICI118551 were pretreated before stimulation of β_1_-AA in cardiac fibroblasts. Results showed that ICI118551 also partially reversed the proliferation effect induced by β_1_-AA (Fig. 6A). And similar to β_1_-AR blocker metoprolol, ICI118551 reduced the increased cAMP induced by β_1_-AA in part (Fig. 6B). However, β_2_-AR expression of cardiac fibroblasts was unchanged after stimulation with β_1_-AA for 24 h (Fig. 6C). Above-mentioned results suggested that β_2_-AR might be involved in proliferation of fibroblasts induced by β_1_-AA.

To explore why β_2_-AR were involved in β_1_-AA mediated fibroblasts proliferation, we examined the binding of β_1_-AA and β_2_-AR by gene transfection, immunoprecipitation and BLItz system. HA-β_1_-AR or flag-β_2_-AR was overexpressed on HEK293 cells by plasmid transfection respectively. In immunoprecipitation experiments, β_1_-AA interacted with β_1_-AR in HEK293 cells which were β_1_-AR-overexpressed (Fig. 6D.a), but not with β_2_-AR in HEK293 cells which were β_2_-AR-overexpressed (Fig. 6D.b). In agreement with above results, BLItz system results displayed that β_1_-AA directly bound to cell lysis of β_1_-AR-overexpessed HEK 293 cells, but not to cell lysis of β_2_-AR-overexpessed HEK 293 cells, which clearly suggested that β_1_-AA bound to β_1_-AR directly but not to β_2_-AR. (Fig. 6D.c,D.d).

### Involvement of p38MAPK and ERK1/2 in β_1_-AA-mediated proliferation in cardiac fibroblasts

**Figure 7:**
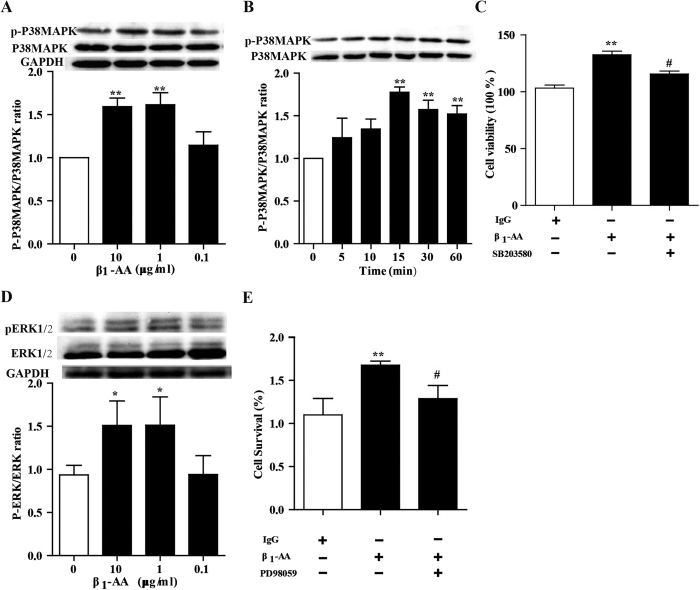
β_1_-AA promoted proliferition in cardiac fibroblast through.activating the P38MAPK and ERK1/2. (**A**) CFs were stimulated with β_1_-AA (0, 0.1, 1, 10 μg/ml) for 30 min, then phospharylated p38MAPK and total p38MAPK were detected by western blot. (**B**) Immunoblot detection of phosphorylated p38MAPK (p-p38MAPK) and total VASP form CFs stimulated with β_1_-AA (10 μg/ml) at 0, 5, 10, 15, 30 and 60 min. (**C**) Cell viability of CFs were pretreated with the P38MAPK inhibitor SB203580 (1 μmol/ml) for 30 min before adding β_1_-AA (10 μg/ml). (**D**) CFs were stimulated with β_1_-AA (0, 0.1, 1, 10 μg/ml) for 30 min, then phospharylated ERK1/2 and total ERK1/2 were detected by western blot. (**E**) Cell viability of CFs were pretreated with the ERK1/2 inhibitor PD98059 (1 μmol/ml) for 30 min before adding β_1_-AA (10 μg/ml). Data shown here are representative of one of three individual experiments with similar results. n = 3 per group. ^**^P < 0.01 *vs.* vehicle (PBS) group.

P38MAPK plays a key role in regulating the proliferation of cardiac fibroblasts. In immunoblot analysis, β_1_-AA resulted in increased p38MAPK phosphorylation in cardiac fibroblasts at 30 min, while total p38MAPK remained unchanged (Fig. 7A). Moreover, phosphorylated p38MAPK began to increase after stimulation of β_1_-AA for 15 min and still increased at 1 h (Fig. 7B). To determine the involvement of p38MAPK in cardiac fibroblasts proliferation, the selective p38MAPK inhibitor SB203580 (1 μmol/L) was used to block the pathway before applied with β_1_-AA (10 μg/ml). As depicted in Fig. 7C, the proliferation of cardiac fibroblasts stimulated by β_1_-AA was partially inhibited by SB203580. These data demonstrated that a role of p38MAPK activation in cardiac fibroblast proliferation was mediated by β_1_-AA. Similarly, β_1_-AA increased phosphorylated ERK1/2 (Fig. 7D), and β_1_-AA was likely to promote the proliferation at 24 h through ERK1/2 as ERK1/2 PD98059 partially reversed the proliferation (Fig. 7E). Taken together, both the activation of p38MAPK and ERK1/2 were involved in the production of cardiac fibroblasts stimulated with β_1_-AA.

### Conditioned medium of cardiac fibroblasts stimulated by β_1_-AA promoted cardiomyocyte early apoptosis

**Figure 8:**
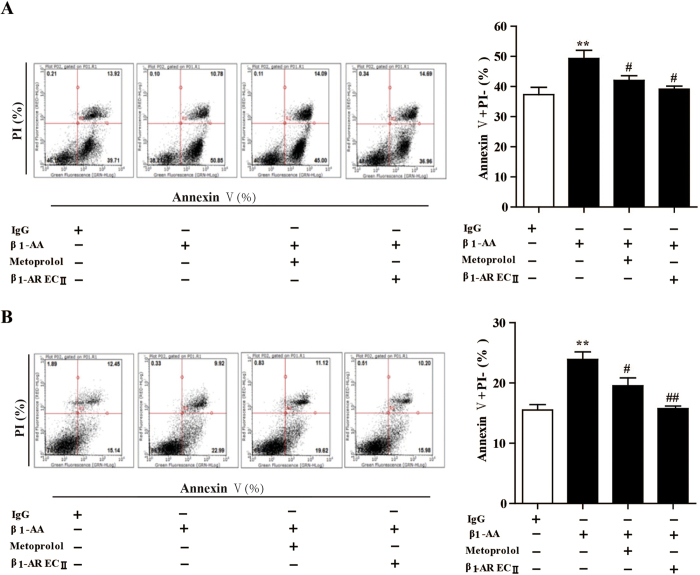
The conditioned medium of cardiac fibroblasts (CFs) treated with β_1_-AA increased early apoptosis in cardiomyocytes. (**A**) The conditioned medium of CFs stimulated with β_1_-AA for 24 h after neutralizing β_1_-AA using β_1_-AR-EC _II_ were added to neonatal rat cardiomyocytes for 24 h, cell early apoptosis was evaluated by Annexin V/PI apoptosis kit. Data shown here are representative of one of three individual experiments with similar results. (**B**) The supernatant of CFs stimulated by β_1_-AA for 24 h promoted the early apoptosis of H9c2 cell lines in 24 h. ^**^P < 0.01 *vs.* conditional medium of CFs treated with IgG group. ^#^*P* < 0.01 *vs.* conditional medium of CFs treated with β_1_-AA group.

To determine whether β_1_-AA promoted the secretion of cardiac fibroblasts, the supernatant of neonatal rat cardiac fibroblasts was harvested after stimulation of β_1_-AA for 24 h and added to cardiomyocytes to detect the cell viability and apoptosis of neonatal rat cardiomyocytes for 24 h. Here β_1_-AA included in the supernatant was neutralized by co-incubation with β_1_-AR-EC _II_ for 1h at 37 °C, in order to rule out the influence of β_1_-AA on cardiomyocyte. As a result, the conditioned medium of β_1_-AA activated cardiac fibroblasts indeed increased early apoptosis of cardiomyocyte and H9c2 cell lines at 24 h detected by an Annexin V/PI apoptosis kit (Fig. 8A,B), which indicated that the conditioned medium of cardiac fibroblasts induced by β_1_-AA promoted early apoptosis of cardiomyocytes, which indicated that β_1_-AA changed the secretion of cardiac fibroblasts.

## Discussion

In the present study we demonstrate that the presence of β_1_-AA directly trigger cardiac dysfunction and collagen deposition in a β_1_-AR monoclonal antibody-positive mouse model. Further, we provide the first evidence that β_1_-AA can promote proliferation in cardiac fibroblasts partially through β_1_-AR, β_2_-AR, P38MAPK and ERK1/2 pathway *in vitro*. Interestingly, β_1_-AA activated the downstream signalling PKA and P38MAPK in 1 h.

A lot of clinical investigations reported that the prevalence of β_1_-AA induced cardiac dysfunction in patients with a variety of cardiovascular diseases^9^. The removal of circulating autoantibodies had beneficial effects on cardiac function of some patients with cardiomyopathy and heart failure^29^. To explore the underlying pathological mechanism of β_1_-AA, a β_1_-AA positive model in mouse seems to be essential. As illustrated in previous studies, β_1_-AA was obtained from sera of β_1_-AA-positive patients^25^ or active β_1_-AR EC_II_ immunized animals^14^. However, it seems that so-called β_1_-AA did not reflect the biological effects of β_1_-AA completely because of the non-specific IgG involved in. Here we obtained a monoclonal antibody against β_1_-AR-EC_II_ using hybridoma technology in order to eliminate the interference of non-specific IgGs. This “synthetic” β_1_-AA bound to β_1_-AR by SA-ELISA and increased the beating rate of neonatal rat cardiomyocytes by activating β_1_-AR, indicating that anti-β_1_-AR monoclonal antibody exhibited functional activity. As a result, we used anti-β_1_-AR monoclonal antibody as β_1_-AA in our studies.

Previously, a β_1_-AA-positive model was created using synthetic peptide corresponding to β_1_-AR-EC_II_^30^. However, such antigens can’t be excluded themselves as pathological causes of myocardial injury on the body. In present study, anti-β_1_-AR monoclonal antibodies were intraperitoneally injected into BALB/c mice to establish a passive β_1_-AA immunized mice model, in which the concentration of β_1_-AA matched that of clinical cardiovascular patients very closely by using SA-ELISA^25^. Cardiac function, in terms of ejection fraction and fractional shortening^31^ in this model was assessed by M-mode ultrasound. Our results showed that β_1_-AA exposure in mice for 16 weeks worsen the systolic function in terms of decreased EF and FS while the diastolic function did not change because of unchanged E/A ratio. Furthermore, it showed the left ventricular dilated change and declined HW/BW in β_1_-AA treated mice, which may indicate that there is a massive of cardiomyocytes death due to cardiomyocytes apoptosis^32^ or autophagy^14^. Additionally, the heart rate did not change during passive β_1_-AA immunization for 16 weeks in the current study (Supplementary material 3). Maybe it should be observed for a longer time, it will be in agreement with the decreased heart rate previously^30^. And we further confirm that β_1_-AA is an independent risk factor of cardiac dysfunction which are consistent with previous reports^33^,^34^. Furthermore, we showed that the expression of α-SMA by immunofluorescence labeling increased in β_1_-AA group as early as the 8^th^ week, which suggested that the phenotypic change of fibroblasts to myofibroblasts. And at the same time, collagen deposition occurred in the mice heart of β_1_-AA group (Supplementary material 2). At the 16^th^ week, we found that the area of collagen deposition in the passive β_1_-AA immunized mouse model by Masson staining was much larger than that in the saline group. These results indicated that the existence of β_1_-AA induced cardiac fibrosis. We believe that β_1_-AA contribute to myocardial fibrosis in patients with heart failure. However, it should be noted that this study has examined only a phenomenon of β_1_-AA mediated cardiac fibrosis.

Proliferation of cardiac fibroblasts is a determinant factor in maintaining cardiac extracellular matrix and fibrotic myocardial remodeling in injured and failing hearts^21^. We found the novel results that cell viability and proliferation index of cardiac fibroblasts were both enhanced by stimulation with β_1_-AA. Then, the problem is how β_1_-AA alone promotes proliferation in cardiac fibroblasts. It has been reported that β_1_-AR/cAMP/PKA signaling is the typical manner of β_1_-AA activating cell. We have previously shown that β_1_-AA promoted proliferation of T lymphocytes via β_1_-adrenocptor/cAMP/PKA pathway. It has been reported that β_1_-AR was expressed in cardiac fibroblasts^27^. In addition, it has been reported that β_1_-AA induced the overexpression of fibroblast CD40 and trigger generation of protaglandin E2^35^,^36^. Here we put forward thatβ_1_-AA activated β_1_-AR in cardiac fibroblasts. Moreover, we showed that β_1_-AA mediated proliferation of cardiac fibroblasts partially via β_1_-AR/cAMP/PKA pathway, which is similar to T lymphocytes proliferation induced by β_1_-AA^25^ while isopposite to β_1_-AA mediated apoptosis of cardiomyocytes^32^.

The remaining problem is what is the other pathway involved in β_1_-AA-induced cardiac fibroblast proliferation. Recent studies implied that β_2_-AR mediated the proliferation of cardiac fibroblasts induced by β_1_/β_2_-adrenoceptor agonist ISO^23^. There are a lot of similarity between β_1_-AR and β_2_-AR on the structure, and they not only have the same Gs pathway but also both cause Gs/Gi switching^37^. For this reason, we assume that the interaction between β_1_-AR and β_2_-AR might be involved in the proliferation of cardiac fibroblasts induced by β_1_-AA. In present study, pretreatment with β_2_-AR selected antagonist ICI118551 partially reversed proliferative effect induced by β_1_-AA. However, β_1_-AA did not change the expression of β_2_-AR in cardiac fibroblasts at 24 h. To our knowledge we this study provides the first evidence indicating that β_2_-AR may participate in the fibroblasts proliferation induced by β_1_-AA. However, these findings were not used to determine how β_1_-AA induced the interaction of β_1_-AR and β_2_-AR.

A large number of publications already described the involvement of MAPK in the induction of proliferation after treatment of various cell types with different mitogens^38^. We previously demonstrated that β_1_-AA was likely to promote proliferation in T lymphocytes through p38MAPK^25^. Here we found that β_1_-AA increased the level of phosphorylated p38MAPK and ERK1/2 in cardiac fibroblast, which is similar to previous study. We have demonstrated that p38MAPK may be involved in the proliferation of cardiac fibroblasts. Besides, β_1_-AA activated ERK1/2^29^ and p38MAPK^24^. Interestingly, β_1_-AA persistently activated p38MAPK in 1 h which indicated that β_1_-AA activated cardiac fibroblast lack of desensitization. This effect is different from that isoprenaline. Recent study reported that the downstream of cAMP Epac mediated MAPK played a significant role in proliferation of cardiac fibroblasts^39^, however, whether activated MAPK induced by β_1_-AA was mediated by Epac, which will be explore in the further study.

Recently, it is reported that β_1_-AA activated β_1_-AR in the neonatal rat cardiomyocytes which lacked desensitization of β_1_-AR^13^. In this study, we determined the downstream signalling of β_1_-AR in cardiac fibroblasts treated with β_1_-AA. We found that β_1_-AA activated PKA and P38MAPK in 1 h. And the endocytosis of β_1_-AR expressed on the surface of HEK 293 cells was lower than in that induced by agonist isoprenaline (data not shown), which is consistent with reported research^40^. Our findings further supported the result β_1_-AA did not induce agonist-induced desensitization^13^, which deserve attention in the future study.

Evidence has shown that, under physiological conditions, cardiac fibroblasts secreted brain natriuretic peptide, extracellular matrix, IL-6, TGF-β, and other compounds that help regulate cardiac function^41^. However, the excessive extracellular matrix deposition contributes to cardiac dysfunction. A previous research suggested β_1_-AA induced fibroblast CD40 and triggered prostaglandin E2 generation^35^. Our results indicated that conditioned medium of cardiac fibroblasts treated with β_1_-AA changed the secretion of cardiac fibroblasts, which promote apoptosis of cardiomyocytes. Here, the conditioned medium including β_1_-AA was neutralized by β_1_-AR EC_II_ to rule out the effects of β_1_-AA on the cardiomyocytes. Results showed that the conditional medium of cardiac fibroblasts promoted apoptosis in cultured neonatal rat cardiomyocytes and H9c2 cardiac cell lines, and the apoptotic baseline of rat neonatal cardiomyocytes was much greater than that of H9c2 cardiac cells. we observed that rat neonatal cardiomyocytes indicated by an Annexin V/PI kit may be more susceptible to early apoptosis than H9c2 cardiac cells, indicating that H9c2 cardiac cells are appropriate for studies of apoptosis. These results suggest that β_1_-AA results in injury to cardiac cells indirectly in a manner of activating fibroblasts. Yet we do not clarify underlying factors that may be involved in the changed secretion of cardiac fibroblasts induced by β_1_-AA, which are critical requirements.

In summary, β_1_-AA-positive passive mice model is generated to study the pathological mechanism of β_1_-AA-mediated cardiovascular diseases. In the model, we show that the long term presence of β_1_-AA alone may contribute to cardiac fibrosis and cardiac dysfunction. *In vitro*, β_1_-AA promoted the proliferation and secretion of cardiac fibroblasts through β_1_-AR/β_2_-Adrenoceptor. Our study will help understand the pathological mechanism of β_1_-AA.

However, β_1_-AA induced the proliferation and secretion of cardiac fibroblasts is just one of the causes in cardiac fibrosis, and our limitation is that we did not supply for pre-treatment of β_1_-AR blocker metoprolol and PKA inhibitor H89 in mice model. Additionally, recent study showed that endothelial dysfunction preceded LV dysfunction related to fibrosis. It has been reported that β_1_-AA induced endothelial dysfunction in conductance and resistance arteries of the wistar rat^42^, which indicated that autoantibody might exert deleterious effect on coronary vasomotricity. However, a lot of experiments need to do in order to clarify the hypothesis and the corresponding mechanisms. And we will study on it in the further study.

In addition, a lot of factors are involved in cardiac fibrosis, such as apoptosis of cardiomyocytes, excessive pressure loading. It has been reported that knockout of β_1_-AR attenuated pressure overload-induced cardiac fibrosis^43^, which suggested that β_1_-AR was involved in the pressure overload-induced fibrosis. Here tail pressure in mice of β_1_-AA group exhibited higher systolic blood pressure as well as unchanged diastolic blood pressure compared with vehicle group (Supplementary material 4). However, whether the increased systolic blood pressure was involved in β_1_-AA induced cardiac fibrosis and the underlying mechanisms needs to be clarified by a lot of experiments in the further study. Additionally, many evidence demonstrated that galectin-3 is closely related to cardiac fibrosis even may be a predictor of heart failure^44^,^45^. However, whether galectin-3 is involved in β_1_-AA mediated cardiac fibrosis is unknown, which may be the new direction of our study.

## Materials and Methods

### Preparation of monoclonal antibodies to β_1_-adrenoceptor

Long peptide (H-W-W-R-A-E-S-D-E-A-R-R-C-Y-N-D-P-K-C-C-D-F-V-T-N-R-C) and short peptide (C-H-W-W-R-A-E-S-D-E-A-R-R) were respectively synthesized according to the amino acid sequence of the second extracellular loop of β_1_-adrenergic receptor (β_1_-AR-EC_II_, 197aa-223aa, H-W-W-R-A-E-S-D-E-A-R-R-C-Y-N-D-P-K-C-C-D-F-V-T-N-R-A, the homology rate among human, mouse and rat is 100%) and were coupled as an antigen for immunizing mice. Hybridoma cell lines secreting β_1_-AR monoclonal antibody (β_1_-AR mAb) were generated by hybridoma technology. β_1_-AR mAb were purified from the ascites of BALB/c mice using a MabTrap kit (Amersham Biosciences). The concentration of β_1_-AR mAb and specificities were determined using a BCA Protein Assay kit (Pierce, Rockford, USA) The binding of β_1_-AR mAb and β_1_-AR was detected by ELISA. And the functional activity of β_1_-AR mAb was tested by neonatal mouse cardiomyocytes beating frequency.

### Generation of β_1_-AA passive immunization model in mice

BALB/c mice used in the experiment were healthy, male, 8-week-old from the Experimental Animal Center of Capital Medical University. Animal experiments were performed in compliance with The Guide for the Care and Use of Laboratory Animals published by the National Institutes of Health (NIH Publication No. 85-23, Revised 1996). The study was approved by the Institutional Animal Care and Use Committee of Capital Medical University and the Guide for the regulation in the People’s Republic of China.

The mice which were sera-negative for β_1_-AA were selected and randomly divided into β_1_-AA positive group and vehicle group. β_1_-AA positive mice model was established by intraperitoneal injection of monoclonal antibodies against β_1_-AR EC_II_ in a dose of 5 μg/g body weight once every two weeks during 16 weeks while the vehicle group was treated in the same way with saline. The sera were obtained in both of the two groups before each treatment. β_1_-AA was measured in sera using Enzyme-linked immunosorbent assay (ELISA) as previously described^30^ and results were expressed as absorbance (*A*) value.

### Echocardiography

For echocardiography, mice were anesthetized with 1.5% isoflurane. Two-dimensional (2D) echocardiographic views of the mid-ventricular short axis were obtained at the level of the papillary muscle tips below the mitral valve (Vevo 770, VisualSonic, Canada). Left ventricular ejection fraction (EF), fractional shortening (FS) and early diastolic and late diastolic mitral flow velocity ratio (E/A Ratio) were calculated by the parameters as the assessment of cardiac function in mice^31^. Left ventricular end-diastolic diameter (LVEDD), left ventricular posterior wall end-diastolic thickness (LVPWT) and left ventricular mass (LV mass) measured by M-mode echocardiography evaluated the structure of left ventricular.

### Masson trichrome

At the end of immunization at 16 weeks, mice of β_1_-AA and vehicle group were killed. Heart tissues were taken from mice quickly and fixed by 4% paraformaldehyde in phosphate buffered saline (PBS) for 48 hours, embedded in paraffin and sectioned (4 μm). Samples were then stained with Masson Trichrome staining for evaluation of collagen deposition. All tissue slides were analyzed by optical microscopy (Olympus BX45, Olympus Corporation, Tokyo, Japan) by the double-blind fashion.

### Immunofluorescence

At the 8^th^ week of immunization, mice of β_1_-AA and vehicle group were killed. Heart tissues were taken from mice quickly and fixed by 4% paraformaldehyde in PBS for 48 hours, embedded in paraffin and sectioned (4 μm). Samples were then stained with immunofluorescence labeling of α-SMA for evaluation of fibrogenic myofibroblasts. Anti-α-SMA primary antibody (1:100 dilution, Abcam, Cat. No. ab5694) was added to the section of heart tissue overnight at 4 °C in the experiment. After three washes with PBS, samples were probed with a fluorescently labeled TRITIC (1:200 dilution, ZSGB-BIO, Cat. No. ZF-0313) secondary antibody for 1 h at room temperature. Following washout, cells were dyed using DAPI and imaged on the fluorescence microscope.

### Cell culture

Cardiac fibroblasts and cardiomyocytes were isolated from neonatal rat (1–3 days old) using trypsin and collagenase II. H9c2 myocardial cell line and HEK 293 cells were from China Infrastruture of Cell Line Resources. Cells were placed into culture media (DMEM, 10% normal bovine serum and 100 U/ml penicillin/streptomycin) and were incubated in incubator with 5% CO_2_ and 95% air at 37 °C. The neonatal rat cardiac fibroblasts and cardiomyocytes were examined for positivity for vimentin and α-actinin respectively. Fibroblasts in passages 3–6 were placed in serum-free-medium for 24 h before each treatment. There were no contaminated cells used in the present study.

### Cardiac fibroblasts proliferation

#### Cell viability assay

Cell viability was measured with a cell counting kit-8 (CCK-8). Cardiac fibroblasts were cultured in either the presence or absence of PBS, β_1_-AA-negative mouse IgG (1 μg/ml, 5 μg/ml, 10 μg/ml, 20 μg/ml), β_1_-AA (1 μg/ml, 5 μg/ml, 10 μg/ml, 20 μg/ml), Isoprenaline (10^−8^mol/L, 10^−7^mol/L 10^−6^mol/L, 10^−5^mol/L), β_1_-AR blocker metoprolol, PKA inhibitor H89 and β_2_-AR blocker ICI118551 in 96-well plate for 24 h. Blockers were added 1 h before β_1_-AA. After each treatment, 10  μl CCK-8 (Dojindo Molecular Technologies, CK04, Kumamoto, Japan) solution was added to each well and the cells were incubated at 37 °C for 2 h in 5% CO_2_ incubator. The absorbance of each well was measured at a wavelength of 450 nm with a microplate reader by the following equation: viability %= [(AS-AB)/(AC-AB)] × 100%, where AS is the absorbance of the samples, AC is the absorbance of the DMEM media, and AB is the absorbance of the control.

#### CFSE labeling and analysis

CFSE is used for cell tracking and proliferation studies. Cardiac fibroblasts were labeled with 5 μmol/L CFSE (Invitrogen Corporation) for 15 min. After the treatments were performed for 24 h, cardiac fibroblasts were harvested and analyzed by flow cytometry. The proliferation index of cardiac fibroblasts was analyzed by Modfit analysis software.

### Binding of β_1_-AA and β_1_-AR

#### Immunofluorescence cellular staining

Cultured cardiac fibroblasts in 24-well-plate which was pretreated with poly-L-lysine were fixed for 30 min at room temprature in 4% paraformaldehyde, followed by three washes with phosphate buffered saline (PBS). Cells were permeabilized with 0.1% TritonX-100 in PBS for 10 minutes and blocked with 5% normal goat serum for 1 h. We used the following primary antibodies in our studies: vimentin (1:200 dilution, Sigma-Aldrich, Cat. No. V6389), α-actinin (1:1000 dilution, Sigma-Aldrich, Cat. No. A7811) and β_1_-adrenergic receptor (1:200 dilution, Abcam, Cat. No. ab3442). After three washes with PBS, samples were probed with a fluorescently labeled TRITIC (1:200 dilution, ZSGB-BIO, Cat. No. ZF-0313) or FITC (1:200 dilution, ZSGB-BIO, Cat. No. ZF-0311) secondary antibody or for 1 h at room temperature. Following washout, cells were dyed using DAPI and imaged on the laser scanning confocal microscope for the cellular identification and expression of β_1_-AR.

#### Protein-protein interaction

The interaction between β_1_-AA and β_1_-AR (or β_2_-AR) was analyzed using the BLItz system (ForteBio, Menlo Park, CA). Non-specific mouse IgG (Sigma-Aldrich. Cat. No. ab3442), purchased primary anti-β_1_-AR (Abcam, Cat. No. ab3442), purchased primary anti-β_2_-AR (Abcam, Cat. No. ab182136) or β_1_-AA attached to protein A/G biosensors respectively (Fortebio). Next, cardiac fibroblasts or HEK 293 cells which were lysed using cell lyse buffer were added to detect the direct binding of cells and above antibodies pre-bound to the protein A/G biosensor. The steps in cardiac fibroblsts were as follows: initial baseline for 120 s, loading for 240 s, baseline for 120 s, association for 240 s, and dissociation for 240 s. The affinity value of the binding of β_1_-AA and β_1_-AR was generated by BLItz pro software analysis.

#### Immunoprecipitation

Cells were lysed in immunoprecipitation buffer. 500 μg of cell lysate was incubated with or without 500 ng/ml Protein A/G (Santa Cruz Biotechnology) at 4 °C for 4 h. Immune complexes were precipitated with the magnetic beads that were pre-bound with appropriate antibodies (IgG, rabbit polyclonal anti-β_1_-AR primary antibodies, rabbit polyclonal anti-β_2_-AR primary antibodies or β_1_-AA) overnight at 4 °C, followed by four washes with the immunoprecipitation buffer. Immune complexes were then eluted from the beads with 2 × SDS sample buffer and subjected to SDS-PAGE.

#### Determination of cAMP production

Cardiac fibroblasts were seeded in 24-well plates at a density of 1 × 10^5^ cells/well. cAMP production of cells was measured after different treatment for 15 min at 37 °C in a humidified incubator in HEPES containing 1 mM 3-isobutyll-methylxanthine (IBMX; Sigma, St. Louis, MO, USA). The cAMP content of the cell lysate was determined by [^125^I] cAMP Radio-Immunoassay KIT according to the manufacturer’s protocol. The assay is based on the competition between unlabelled cAMP and fixed quantity of [^125^I] labelled cAMP-specific antibody, and the cell-associated radioactivity was counted in a 1470 Wallac Wizard γ counter (Perkin Elmer).

#### Western blot analysis

To perform PKA activity, phospho-vasp^Ser 157^ and total-vasp were analyzed by western blot^25^, after a rapid rinse with PBS, cardiac fibroblasts were scraped from 6-well multi-well plates, lysed with RIPA buffer supplemented with protease inhibitor and phosphatase inhibitor. Total protein concentration in each sample was measured by the BCA assay using bovine serum albumin as the standard protein. A fixed amount of protein (30 μg) from each sample was fractionated in 12% SDS-polyacrylamide gels by electroresis and then transferred to immobilon-FL PVDF membranes (Millipore, IPVH00010). Blots were probed using rabbit anti-phospho-vasp ^Ser 157^ (1: 1000, Cell Signaling Technology, Cat. No. 3111) or anti-total-vasp antibodies (1:1000, Cell Signaling Technology, Cat. No. 3112). Mouse anti-GAPDH (1:1000, Sigma-aldrich, Cat. No. SAB1405848) or β-actin (1:1000, Cell Signaling Technology, Cat. No. 12620) was used as protein loading control. All bands were normalized to their respective controls.

#### Transfection

HEK293 cells plated in a 6-well plate were transfected with HA-β_1_-AR or flag-β_2_-AR plasmid DNA using Lipofectamine 2000 transfection reagent (Invitrogen, Cat. No. 11668019) following the manufacturer’s recommendations. Experiments were performed 24 h after the transfection.

#### Assessment of cell apoptosis

Conditioned medium of different treatment of cardiac fibroblast for 24 h was added to NRCMs and H9c2 myocardial cells for 24 h. NRCMs and H9c2 were harvested, washed and re-suspended in binding buffer 24 h after stimulation. The apoptosis of cells was determined with an FITC Annexin V/PI apoptosis detection kit according to the instructions of the manufacturer. Briefly, cells were washed three times in PBS and subsequently in the dark in 200 μl of binding buffer containing 10 μl Annexin V- FITC and 5 μl of propidium iodide (PI). Afterward, cell apoptosis was analyzed by flow cytometry within 1 h.

#### Statistics

All statistical analysis was performed using of SPSS 15.0 statistical software. One way-ANOVA followed by post hoc Bonferroni correction was performed on proliferation of fibroblasts among different groups. All the experimental data were expressed as mean ± standard error. Repeated measurement data analysis of variance was using for continuous measurement of the experimental data at different time points, probabilities of 0.05 or less was considered statistically significant.

## Additional Information

**How to cite this article**: Lv, T. *et al*. Proliferation in cardiac fibroblasts induced by β_1_-adrenoceptor autoantibody and the underlying mechanisms. *Sci. Rep.* **6**, 32430; doi: 10.1038/srep32430 (2016).

## Supplementary Material

Supplementary Information

## References

[b1] Kong P., Christia P. & Frangogiannis N. G. The Pathogenesis of Cardiac Fibrosis. Cellular and Molecular Life Sciences 71, 549–574, doi: 10.1007/s00018-013-1349-6 (2014). 23649149 PMC3769482

[b2] Perazzolo Marra M. . Impact of the presence and amount of myocardial fibrosis by cardiac magnetic resonance on arrhythmic outcome and sudden cardiac death in nonischemic dilated cardiomyopathy. Heart rhythm: the official journal of the Heart Rhythm Society 11, 856–863, doi: 10.1016/j.hrthm.2014.01.014 (2014). 24440822

[b3] Fu Y., Xiao H. & Zhang Y. Beta-adrenoceptor signaling pathways mediate cardiac pathological remodeling. Frontiers in Bioscience (Elite Edition) 4, 1625–1637 (2012). 22201979 10.2741/484

[b4] Chan V., Fenning A., Hoey A. & Brown L. Chronic beta-adrenoceptor antagonist treatment controls cardiovascular remodeling in heart failure in the aging spontaneously hypertensive rat. Journal of cardiovascular pharmacology 58, 424–431, doi: 10.1097/FJC.0b013e3182283c78 (2011). 21709583

[b5] Seeland U. . Interstitial remodeling in beta1-adrenergic receptor transgenic mice. Basic research in cardiology 102, 183–193, doi: 10.1007/s00395-006-0635-y (2007). 17122889 PMC2779411

[b6] Liu H. R., Zhao R. R., Zhi J. M., Wu B. W. & Fu M. L. Screening of serum autoantibodies to cardiac beta1-adrenoceptors and M2-muscarinic acetylcholine receptors in 408 healthy subjects of varying ages. Autoimmunity 29, 43–51 (1999). 10052684 10.3109/08916939908995971

[b7] Elies R. . Structural and functional analysis of the B cell epitopes recognized by anti-receptor autoantibodies in patients with Chagas’ disease. Journal of immunology (Baltimore, Md.: 1950) 157, 4203–4211 (1996). 8892658

[b8] Holthoff H. P. . Detection of anti-beta1-AR autoantibodies in heart failure by a cell-based competition ELISA. Circulation research 111, 675–684, doi: 10.1161/circresaha.112.272682 (2012). 22811559

[b9] Aso S. . Anti-beta 1-adrenoreceptor autoantibodies and myocardial sympathetic nerve activity in chronic heart failure. International journal of cardiology 131, 240–245, doi: 10.1016/j.ijcard.2007.10.029 (2009). 18199508

[b10] Galloway A. . Activating autoantibodies to the beta1/2-adrenergic and M2 muscarinic receptors associate with atrial tachyarrhythmias in patients with hyperthyroidism. Endocrine 49, 457–463, doi: 10.1007/s12020-014-0495-4 (2015). 25500789 PMC5810549

[b11] Christ T. . Autoantibodies against the beta1 adrenoceptor from patients with dilated cardiomyopathy prolong action potential duration and enhance contractility in isolated cardiomyocytes. Journal of molecular and cellular cardiology 33, 1515–1525, doi: 10.1006/jmcc.2001.1414 (2001). 11448139

[b12] Chiale P. A. . Differential profile and biochemical effects of antiautonomic membrane receptor antibodies in ventricular arrhythmias and sinus node dysfunction. Circulation 103, 1765–1771 (2001). 11282908 10.1161/01.cir.103.13.1765

[b13] Magnusson Y., Wallukat G., Waagstein F., Hjalmarson A. & Hoebeke J. Autoimmunity in idiopathic dilated cardiomyopathy. Characterization of antibodies against the beta 1-adrenoceptor with positive chronotropic effect. Circulation 89, 2760–2767 (1994). 8205690 10.1161/01.cir.89.6.2760

[b14] Wang L. . Decreased autophagy: a major factor for cardiomyocyte death induced by beta1-adrenoceptor autoantibodies. Cell death & disease 6, e1862, doi: 10.1038/cddis.2015.237 (2015). 26313913 PMC4558518

[b15] Wallukat G. . Agonist-like beta-adrenoceptor antibodies in heart failure. The American journal of cardiology 83, 75H–79H (1999). 10.1016/s0002-9149(99)00265-9 10750592

[b16] Li H. . Inducible cardiac arrhythmias caused by enhanced β1-adrenergic autoantibody expression in the rabbit. American journal of physiology. Heart and circulatory physiology 306, H422–428, doi: 10.1152/ajpheart.00551.2013 (2014). 24271491 PMC3920144

[b17] Zuo L. . Pro-arrhythmic action of autoantibodies against the second extracellular loop of beta1-adrenoceptor and its underlying molecular mechanisms. International journal of cardiology 198, 251–258, doi: 10.1016/j.ijcard.2015.06.144 (2015). 26241168

[b18] Iwata M. . Autoantibodies against the second extracellular loop of beta1-adrenergic receptors predict ventricular tachycardia and sudden death in patients with idiopathic dilated cardiomyopathy. Journal of the American College of Cardiology 37, 418–424 (2001). 11216956 10.1016/s0735-1097(00)01109-8

[b19] Pei J. . The predictive values of beta1-adrenergic and M2 muscarinic receptor autoantibodies for sudden cardiac death in patients with chronic heart failure. European journal of heart failure 14, 887–894, doi: 10.1093/eurjhf/hfs082 (2012). 22713286

[b20] Liu H. R., Zhao R. R., Jiao X. Y., Wang Y. Y. & Fu M. Relationship of myocardial remodeling to the genesis of serum autoantibodies to cardiac beta(1)-adrenoceptors and muscarinic type 2 acetylcholine receptors in rats. Journal of the American College of Cardiology 39, 1866–1873 (2002). 12039504 10.1016/s0735-1097(02)01865-x

[b21] MacKenna D., Summerour S. R. & Villarreal F. J. Role of mechanical factors in modulating cardiac fibroblast function and extracellular matrix synthesis. Cardiovascular research 46, 257–263 (2000). 10773229 10.1016/s0008-6363(00)00030-4

[b22] Leicht M., Briest W. & Zimmer H. G. Regulation of norepinephrine-induced proliferation in cardiac fibroblasts by interleukin-6 and p42/p44 mitogen activated protein kinase. Molecular and cellular biochemistry 243, 65–72 (2003). 12619890 10.1023/a:1021655023870

[b23] Chen C. .β-Adrenergic receptors stimulate interleukin-6 production through Epac-dependent activation of PKCδ/p38 MAPK signalling in neonatal mouse cardiac fibroblasts. British journal of pharmacology 166, 676–688, doi: 10.1111/j.1476-5381.2011.01785.x (2012). 22103274 PMC3417497

[b24] Liu N. . HIP-55/DBNL-dependent regulation of adrenergic receptor mediates the ERK1/2 proliferative pathway. Molecular bioSystems 10, 1932–1939, doi: 10.1039/c3mb70525k (2014). 24802081

[b25] Du Y. . beta1-Adrenoceptor autoantibodies from DCM patients enhance the proliferation of T lymphocytes through the beta1-AR/cAMP/PKA and p38 MAPK pathways. PloS one 7, e52911, doi: 10.1371/journal.pone.0052911 (2012). 23300817 PMC3534136

[b26] Tutor A. S., Penela P. & Mayor F. Jr. Anti-beta1-adrenergic receptor autoantibodies are potent stimulators of the ERK1/2 pathway in cardiac cells. Cardiovascular research 76, 51–60, doi: 10.1016/j.cardiores.2007.05.022 (2007). 17628514

[b27] Lai K. B., Sanderson J. E. & Yu C. M. Suppression of collagen production in norepinephrine stimulated cardiac fibroblasts culture: differential effect of alpha and beta-adrenoreceptor antagonism. Cardiovascular drugs and therapy / sponsored by the International Society of Cardiovascular Pharmacotherapy 23, 271–280, doi: 10.1007/s10557-009-6183-6 (2009). 19575289

[b28] Calebiro D. . Persistent cAMP-Signals Triggered by Internalized G-Protein–Coupled Receptors. PLoS Biology 7, doi: 10.1371/journal.pbio.1000172 (2009). PMC2718703 19688034

[b29] Jahns R. . Autoantibodies activating human beta1-adrenergic receptors are associated with reduced cardiac function in chronic heart failure. Circulation 99, 649–654 (1999). 9950662 10.1161/01.cir.99.5.649

[b30] Zuo L. . Long-term active immunization with a synthetic peptide corresponding to the second extracellular loop of beta1-adrenoceptor induces both morphological and functional cardiomyopathic changes in rats. International journal of cardiology 149, 89–94, doi: 10.1016/j.ijcard.2009.12.023 (2011). 20096470

[b31] Lopez-Andres N. . Cardiotrophin 1 is involved in cardiac, vascular, and renal fibrosis and dysfunction. Hypertension 60, 563–573, doi: 10.1161/hypertensionaha.112.194407 (2012). 22733458

[b32] Jane-wit D. . Beta 1-adrenergic receptor autoantibodies mediate dilated cardiomyopathy by agonistically inducing cardiomyocyte apoptosis. Circulation 116, 399–410, doi: 10.1161/circulationaha.106.683193 (2007). 17620508

[b33] Wang L. . Decreased autophagy in rat heart induced by anti-beta1-adrenergic receptor autoantibodies contributes to the decline in mitochondrial membrane potential. PloS one 8, e81296, doi: 10.1371/journal.pone.0081296 (2013). 24278413 PMC3835737

[b34] Segovia M., Reina S., Borda E. & Sterin-Borda L. Autoantibodies to the beta(1)-Adrenoceptor from Patients with Periodontitis as a Risk Factor for Cardiac Dysfunction. ISRN dentistry 2011, 791393, doi: 10.5402/2011/791393 (2011). 21991485 PMC3170702

[b35] Sterin-Borda L., Furlan C. & Borda E. Autoantibodies to beta1-adrenoceptors in human chronic periodontitis induce overexpression of fibroblast CD40 and trigger prostaglandin E2 generation. Journal of periodontal research 44, 330–337, doi: 10.1111/j.1600-0765.2008.01139.x (2009). 18973525

[b36] Sterin-Borda L., Segovia M., Reina S. & Borda E. beta1-Adrenoceptor antibody-induced increase in soluble CD40 ligand release in chronic periodontitis patients: role of prostaglandin E(2). Experimental physiology 97, 1030–1039, doi: 10.1113/expphysiol.2012.065748 (2012). 22523383

[b37] Martin N. P., Whalen E. J., Zamah M. A., Pierce K. L. & Lefkowitz R. J. PKA-mediated phosphorylation of the beta1-adrenergic receptor promotes Gs/Gi switching. Cellular signalling 16, 1397–1403, doi: 10.1016/j.cellsig.2004.05.002 (2004). 15381255

[b38] Yu M., Zheng Y., Sun H. X. & Yu D. J. Inhibitory effects of enalaprilat on rat cardiac fibroblast proliferation via ROS/P38MAPK/TGF-beta1 signaling pathway. Molecules (Basel, Switzerland) 17, 2738–2751, doi: 10.3390/molecules17032738 (2012). 22395404 PMC6268937

[b39] Chen C. . β-Adrenergic receptors stimulate interleukin-6 production through Epac-dependent activation of PKCδ/p38 MAPK signalling in neonatal mouse cardiac fibroblasts. British journal of pharmacology 166, 676–688, doi: 10.1111/j.1476-5381.2011.01785.x (2012). 22103274 PMC3417497

[b40] Bornholz B. . Impact of human autoantibodies on β1-adrenergic receptor conformation, activity, and internalization. Cardiovascular research 97, 472–480, doi: 10.1093/cvr/cvs350 (2013). 23208588 PMC3567785

[b41] Crawford J. R., Haudek S. B., Cieslik K. A., Trial J. & Entman M. L. Origin of developmental precursors dictates the pathophysiologic role of cardiac fibroblasts. Journal of cardiovascular translational research 5, 749–759, doi: 10.1007/s12265-012-9402-7 (2012). 22972312 PMC3518725

[b42] Abdelkrim M. A. . Antibodies against the second extracellular loop of beta1-adrenergic receptors induce endothelial dysfunction in conductance and resistance arteries of the Wistar rat. International immunopharmacology 19, 308–316, doi: 10.1016/j.intimp.2014.01.029 (2014). 24530918

[b43] Kiriazis H. . Knockout of beta(1)- and beta(2)-adrenoceptors attenuates pressure overload-induced cardiac hypertrophy and fibrosis. British journal of pharmacology 153, 684–692, doi: 10.1038/sj.bjp.0707622 (2008). 18193078 PMC2259198

[b44] Ho J. E. . Galectin-3, a Marker of Cardiac Fibrosis, Predicts Incident Heart Failure in the Community. Journal of the American College of Cardiology 60, 1249–1256, doi: 10.1016/j.jacc.2012.04.053 (2012). 22939561 PMC3512095

[b45] Bošnjak I., Selthofer-Relatić K. & Včev A. Prognostic Value of Galectin-3 in Patients with Heart Failure. Disease Markers 2015, doi: 10.1155/2015/690205 (2015). PMC4415488 25960597

