# Supplementary material for: Proliferation in cardiac fibroblasts induced by β1-adrenoceptor autoantibody and the underlying mechanisms
Source: Sci Rep. 2016 Aug 31;6:32430. doi: 10.1038/srep32430 (PMC5006240; doi:10.1038/srep32430)
Supplement: Supplementary Information [file srep32430-s1.pdf]

Proliferation in cardiac fibroblasts induced by β_1-adrenoceptor autoantibody and the underlying mechanisms

Tingting Lv[a], Yunhui Du[a,b], Ning Cao[a], Suli Zhang[a,b], Yulin Gong[a], Yan Bai[a], Wen Wang[a,b], Huirong Liu[a,b]*

[a] Department of Physiology and Pathophysiology, School of Basic Medical Sciences, Capital Medical University, Beijing 100069, PR China

[b] Beijing Key Laboratory of Metabolic Disorders Related Cardiovascular Disease, Capital Medical University, Beijing 100069, PR China

*Correspondence to: Prof. Huirong Liu, Department of physiology and pathophysiology, School of Basic Medical Sciences, Capital Medical University, Beijing 100069, PR China. Telephone: 011-86-10-83911830; Fax: 011-86-10-63294027; E-mail address: liuhr2000@126.com.

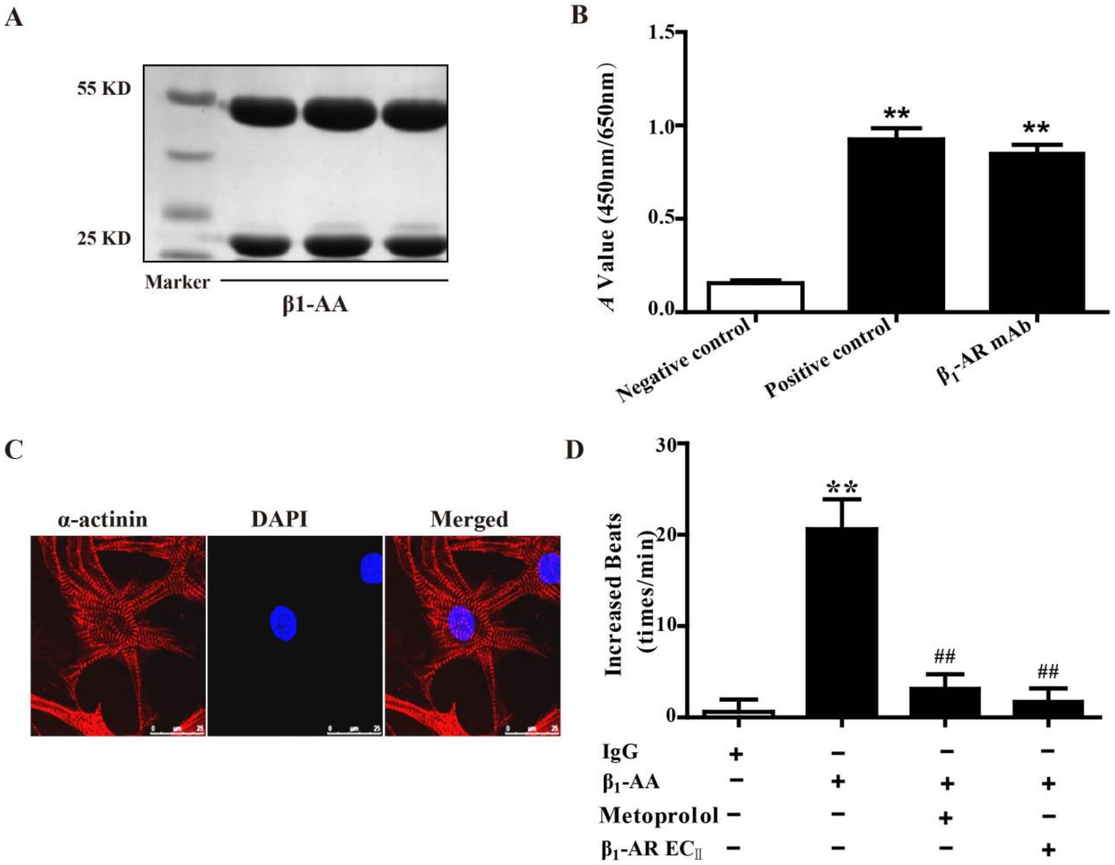

Fig.S1. Functional identification of monoclonal antibodies of β_1-AR EC_{II} (β_1-AR mAb). A. The purity of β_1-AR mAb was detected by SDS-Page. B. The binding of β_1-AR mAb and β_1-AR EC_{II} was detected by SA-ELISA. C. The cultured neonatal rat cardiomyocytes were identified by immunofluorescence cellular staining of α-actinin. D. The increased beating frequency of primary neonatal rat cardiomyocytes was counted to evaluate the activity of β_1-AR mAb. $^{**}P$ <0.01 *vs.* negative control or IgG, $^{\#\#}P$ <0.01 *vs.* β_1-AA, n=6 per group. Data were presented as mean ± SEM of 3 independent experiments.

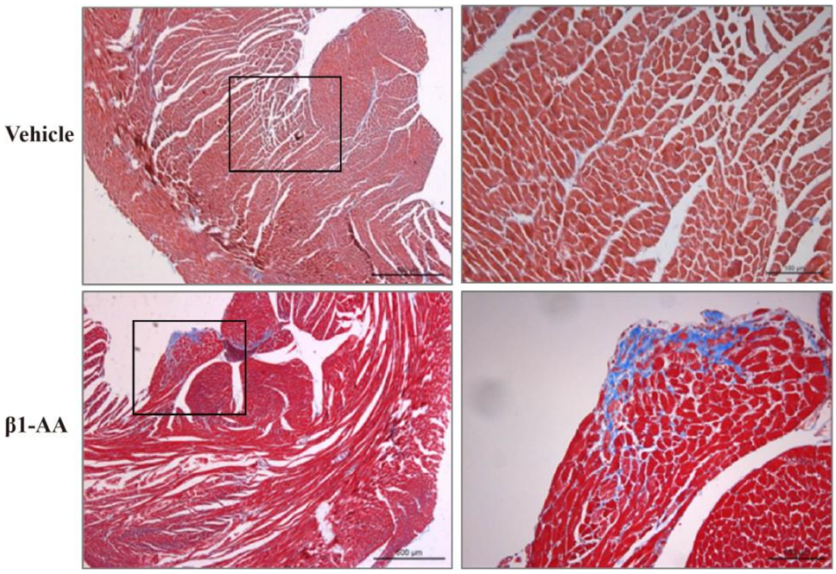

Fig.S2. Collagen deposition was evaluated by Masson staining in the heart of mice in vehicle and β_1-AA group at the 8th week of model. Representative Masson staining of heart tissue of vehicle and β_1-AA group was shown in the figure. The collagen deposition appeared blue. The results showed that the area of collagen deposition around the cardiac cells in β_1-AA group was much more than that in vehicle group. The graph on the right is the enlargement of the left. Scale =500 μm (left), 100μm (right).

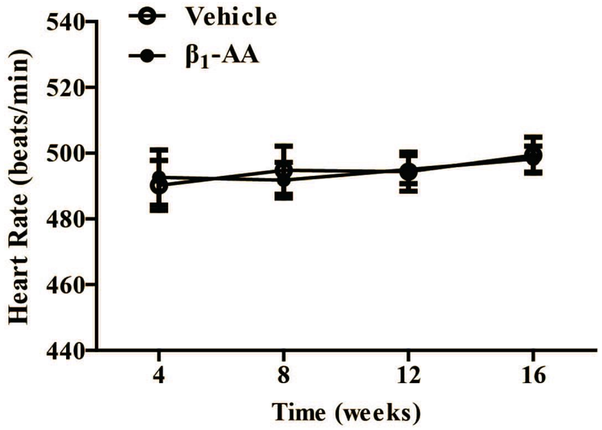

Fig.S3. The characterization of heart rate of mice in vehicle or β_1-AA group during the 16th week. The heart rate of mice in β_1-AA and vehicle group was detected at the 4th, 8th, 12th and 16th during the 16th week. The results showed that there was no obvious difference between β_1-AA and vehicle group. n=8.

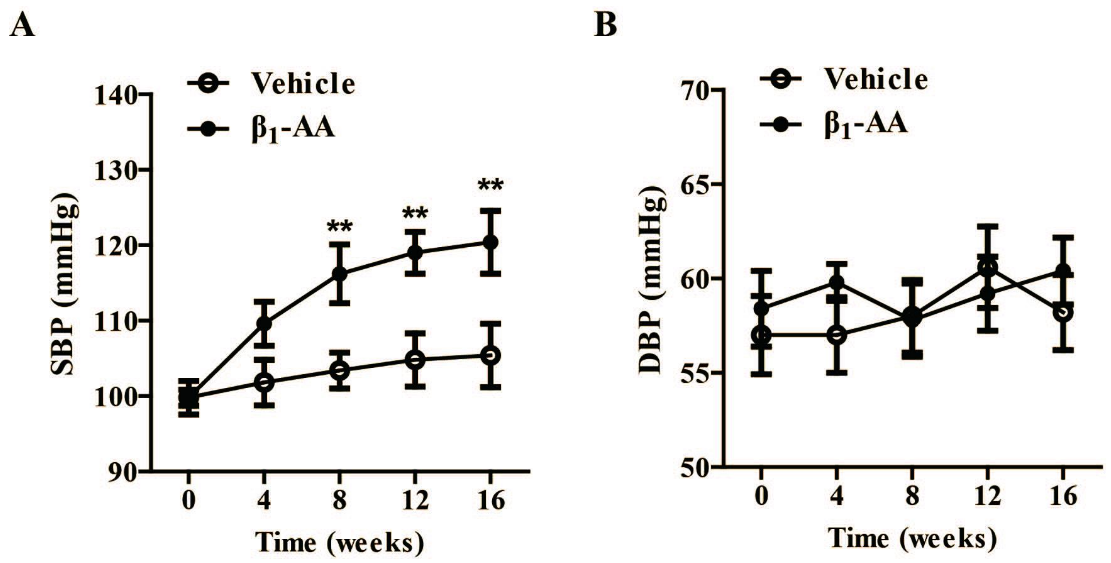

Fig.S4. The dynamic change of blood pressure of mice during the 16th week. A. The systolic blood pressure (SBP) of mice in β_1-AA and vehicle group during the 16th week. B. The diastolic blood pressure (DBP) of mice in β_1-AA and vehicle group during the 16th week. $^{**}P$ <0.01 *vs.* Vehicle group, n=8 per group. Data were presented as mean±SEM of 3 independent experiments.

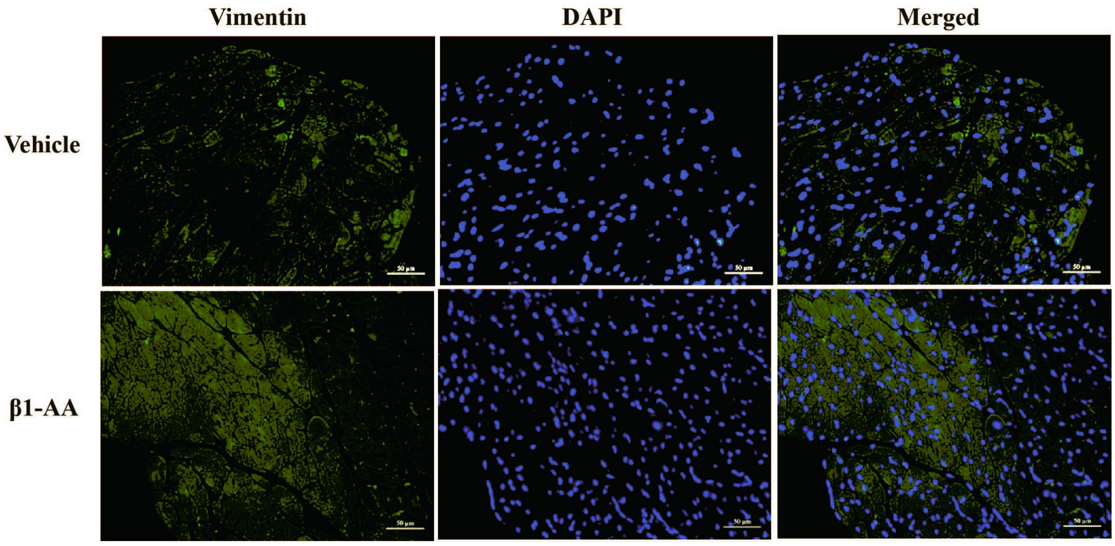

Fig. S5. Cardiac fibroblast proliferated in the heart of mice treated with β_1-AA at the 8th week of passive immunization. The expression of vimentin as the marker of cardiac fibroblasts in heart of β_1-AA group or vehicle group was detected by immunofluorescence labeling, which was observed under the fluorescence microscope. The expression of vimentin in papillary muscle of heart in β_1-AA group was much higher than that in vehicle group, which indicated that the cardiac fibroblasts proliferated after β_1-AA treatment. Scale bar=50 μm.

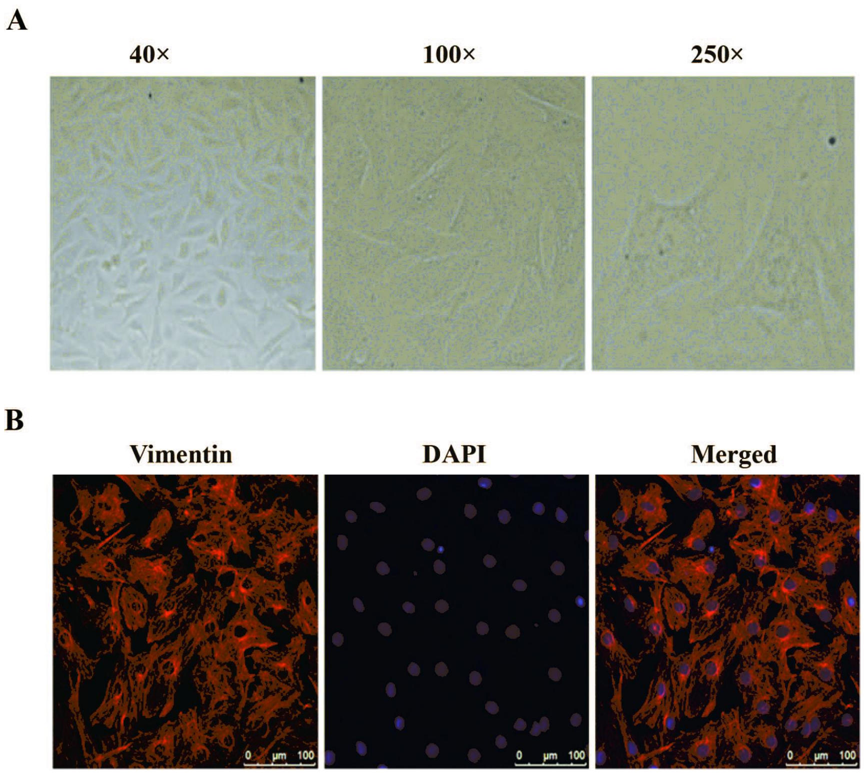

Fig.S6. The positivity of cardiac fibroblasts identified by vimentin was more than 95%. A. The cellular morphology of primary neonatal rat cardiac fibroblasts was observed in light microscope. The magnification is 40, 100 or 250 times. B. The identification of cardiac fibroblasts was performed by immunofluorescence cellular staining of vimentin. The Scale Bar=100 μm.
